# Maternal environmental enrichment affects the corpora lutea and progesterone levels in pregnant mice

**DOI:** 10.3389/fendo.2025.1483893

**Published:** 2025-02-10

**Authors:** Fernanda Luz De la Cruz Borthiry, Jimena Soledad Beltrame, Julieta Aylen Schander, Aime Florencia Silva, Fernanda Parborell, Ana María Franchi, María Laura Ribeiro

**Affiliations:** ^1^ Laboratorio de Fisiología y Farmacología de la Reproducción, Centro de Estudios Farmacológicos y Botánicos, Universidad de Buenos Aires (UBA)-Consejo Nacional de Investigaciones Científicas y Técnicas (CONICET), Ciudad Autónoma de Buenos Aires, Argentina; ^2^ Laboratorio de Fisiopatología de la Preñez y el Parto, Centro de Estudios Farmacológicos y Botánicos, Universidad de Buenos Aires (UBA)-Consejo Nacional de Investigaciones Científicas y Técnicas (CONICET), Ciudad Autónoma de Buenos Aires, Argentina; ^3^ Laboratorio de Fisiopatología Ovárica, Centro de Estudios Farmacológicos y Botánicos, Universidad de Buenos Aires (UBA)-Consejo Nacional de Investigaciones Científicas y Técnicas (CONICET), Ciudad Autónoma de Buenos Aires, Argentina; ^4^ Laboratorio de Estudios de la Fisiopatología del Ovario, Instituto de Biología y Medicina Experimental, Consejo Nacional de Investigaciones Científicas y Técnicas (CONICET)-Fundación Instituto de Biología y Medicina Experimental (IBYME), Ciudad Autónoma de Buenos Aires, Argentina

**Keywords:** maternal enriched environment, gestation, ovary, progesterone, corpora lutea

## Abstract

**Introduction:**

Maternal lifestyle impacts reproductive performance. Previously, we demonstrated that maternal environmental enrichment promotes pregnancy success in BALB/c mice. As progesterone regulates gestation, we decided to study the effect of maternal environmental enrichment on ovarian physiology during early gestation.

**Methods:**

For this, six-week-old female mice were housed in enriched or control cages for six weeks and then mated with control fertile males. Females with a mucus plug were returned to their respective control or enriched cages. Pregnant mice were euthanized on day 7 of pregnancy, and ovaries and progesterone levels were investigated.

**Results:**

Hematoxylin and eosin slices showed no differences in the area (μm^2^) of the ovaries between control and enriched females. Also, the number of primordial, primary, preantral, antral, and atretic follicles was similar for both treatments. However, the number and area (μm^2^) of corpora lutea were increased in the ovaries from the enriched group. Moreover, enriched females presented higher progesterone serum levels and increased 3β-HSD expression.

**Discussion:**

Therefore, maternal environmental enrichment regulates ovarian physiology, and this could promote the benefits previously reported.

## 1 Introduction

The corpus luteum is a transient ovarian endocrine gland generated after ovulation. It produces progesterone, necessary for pregnancy establishment and maintenance. In humans, once it has developed, the placenta becomes the primary source of progesterone. In contrast, the corpus luteum is the main producer of progesterone throughout gestation in rodents (1, 2). Progesterone exerts a crucial role during pregnancy by regulating different processes including the differentiation of the endometrium, the quiescence of the myometrium and the modulation of the immune system (3). Therefore, alterations in the levels of progesterone are associated with difficulties in conceiving and an increased risk of early pregnancy loss or miscarriage (4, 5).

Several studies have shown that lifestyle significantly influences both physiological and pathological events. Moreover, the perinatal environment could impact the quality of gestation, as well as fetal and offspring health (6). In particular, we reported that exposing mice to an enrichment protocol which includes social, physical, and sensory stimuli that enhance their natural behavior, has a beneficial effect on gestation (7). We observed that maternal environmental enrichment regulates uterine physiology, promotes vascular remodeling during early gestation, and prevents pregnancy loss in mice (7). Furthermore, we demonstrated that enrichment of the maternal environment prevents preterm birth in mice, and improves offspring’s health (8, 9). Also, other authors informed that environmental enrichment affects the placental response to stress mediators and protects the fetus against an inflammatory stimulus (10). Altogether, this evidence suggests that modifying the maternal environment could improve pregnancy outcome.

Based on our previous results, and considering that many uterine processes are regulated by ovarian activity, we propose that maternal enrichment could also modulate ovarian physiology. We hypothesize that environmental enrichment may enhance oocyte development or the activity of corpora lutea, thereby contributing to improved pregnancy success. Therefore, we decided to study the effect of maternal environmental enrichment on ovarian morphology and progesterone production during early gestation.

## 2 Materials and methods

### 2.1 Ethical statement

The present study was performed under the recommendations in the Guide for the Care and Use of Laboratory Animals of the National Institutes of Health. The Committee on the Ethics of Animal Experiments of the School of Medicine, University of Buenos Aires (CICUAL; permit number 2547/2019) approved this protocol. Animals were obtained from Bio Fucal S.A. (Buenos Aires, Argentina).

### 2.2 Animals

Six-week-old BALB/c female mice were randomly assigned to either a control or an enriched environment protocol as previously described (7), with 10 animals per group. Control cages (43 × 27 × 17 cm) housed 3 to 4 animals. The enriched cage was larger (64 × 42 × 20 cm) than the control cage and housed 10 females to promote social interaction. The enriched cage contained stairs, tunnels, shelters, running wheels, and other toys made from different materials, which were replaced and rearranged weekly. Additionally, the feeding box was relocated each week to stimulate foraging and exploratory behaviors. This protocol combines non-invasive visual, cognitive, and social stimuli with voluntary physical activity. The animals were housed in control or environmentally enriched cages for 6 weeks. Afterward, females were mated with fertile males of the same strain housed under control conditions. The morning when the vaginal plug was detected was considered day 0 of pregnancy and the females were returned to their respective control or enriched cage. Pregnant animals were euthanized on day 7 of gestation by decapitation. Whole blood was collected and the ovaries were extracted.

### 2.3 Ovarian histology

Ovaries were fixed in Bouin solution overnight, dehydrated in ethanol, and embedded in paraffin. Slices of 5 µm were obtained and separated by 50 µm intervals to avoid counting the same follicle twice. Slices were stained with hematoxylin and eosin to analyze and classify the follicles and corpora lutea under a light microscope (Nikon Eclipse 200).

The structures were classified according to previously established criteria (11, 12) into the following categories: primordial, primary, preantral, antral, and atretic follicles and corpora lutea. The number of follicles and corpora lutea was determined in five sections per ovary and one ovary per animal was analyzed.

Primordial follicles were defined as intact oocytes surrounded by a single layer of flattened follicular cells. Primary follicles presented one layer of cuboidal granulosa cells. Preantral follicles presented two or more layers of granulosa cells and no apparent space between granulosa cells. Antral follicles were characterized by presenting several layers of granulosa cells and the antral cavity. Corpora lutea were characterized by comprising luteal cells, endothelial cells, and immune cells. Atretic follicles were defined as those that exhibited degeneration and detachment of the granulosa cell layer, pyknotic nuclei, and oocyte degeneration. Data are expressed as the total number of each follicle type or corpus luteum counted per ovary. The area of the ovary (µm^2^) and each corpus luteum (µm^2^) was measured using Image-Pro Plus software (version 4.5.0.29, Scion Corporation, Media Cybernetics, Rockville, MD, USA). The corpus luteum area was normalized to the area of the ovary. The mean corpora lutea area per animal was calculated.

### 2.4 Progesterone serum levels measurement

Mice were decapitated and whole blood was collected, allowed to clot for 30 min at room temperature, and then centrifuged at 8000 g for 5 min to obtain serum. The progesterone levels were determined by immunochemiluminescence assay (Biomedical laboratory Dr. Rapela, Buenos Aires, Argentina).

### 2.5 Western blot analysis

Western blot was performed as previously described (7) with minor modifications. One ovary per animal was used. Sixty μg of protein were loaded in each lane and separated in 12,5% SDS-PAGE. The antibodies used were: anti-3β-hydroxysteroid dehydrogenase (3β-HSD) (1:200, Santa Cruz Biotechnologies, catalog #sc-28206), anti-steroidogenic acute regulatory protein (StAR) (1:1000, Novus Biologicals, catalog #NBP1-33485), and peroxidase-conjugated goat anti-rabbit antibody (1:7500, Jackson ImmunoResearch, code 111-035-003). Results were expressed as the relative optical density to total transferred protein determined with Ponceau S Staining Solution.

### 2.6 Statistical analysis

Data was analyzed with t-tests or Mann-Whitney tests using the statistical software Infostat (Facultad de Ciencias Agropecuarias, Universidad de Córdoba, Argentina). Normality and homoscedasticity were assessed using the Shapiro–Wilk and Levene tests, respectively. Mann-Whitney tests were conducted when the data exhibited non-homoscedasticity. One ovary per animal was used for each determination. Four to six mice were used for histological analysis and progesterone quantification, and six to seven for protein studies. Differences were considered statistically significant when the p-value was equal to or less than 0.05.

## 3 Results

**Figure 1:**
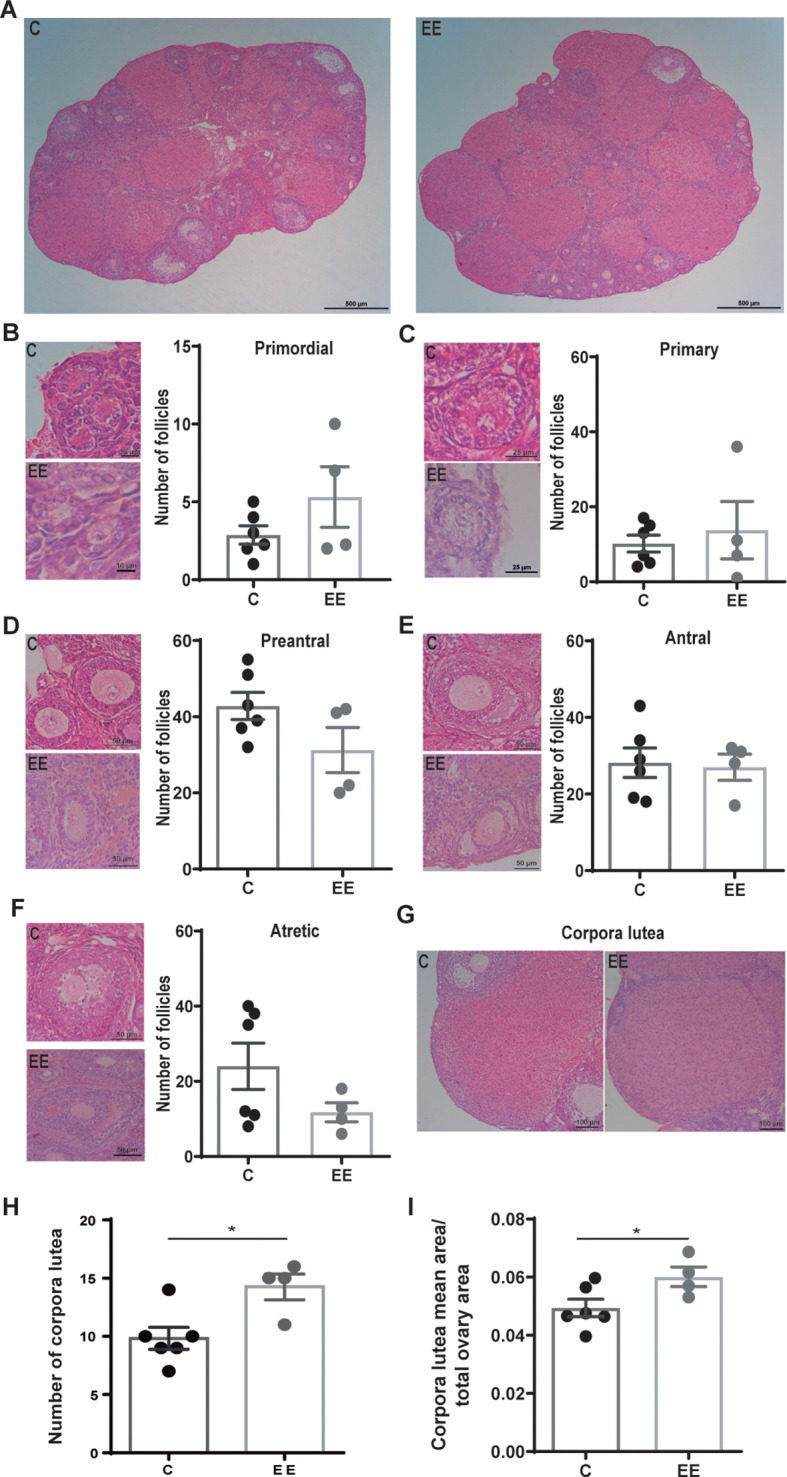
Histological analysis of the ovaries. **(A)** Representative images of d7 ovaries from control and enriched environment animals. Representative images and the number of **(B)** primordial, **(C)** primary, **(D)** preantral, **(E)** antral, and **(F)** atretic follicles. **(G)** Representative images of the corpora lutea. **(H)** Number of corpora lutea per ovary. **(I)** The mean area of corpora lutea normalized to the total area of the ovary. Data are expressed as the total number of follicles or corpora lutea per ovary. The area represents the average area of corpora lutea per animal. One ovary per animal was analyzed. N= 6 control and 4 enriched environment animals. Results are expressed as mean ± s.e.m. T-test was performed for the **(D-F, H, I)** panels. Mann-Whitney test was performed for **(B, C)** panels. *Significant statistical differences. C, control; EE, enriched environment.

First, the effect of maternal environmental enrichment on ovarian structure and size was analyzed. For this, the ovaries were extracted and sectioned for histological studies. The structure of the ovary was conserved in both experimental groups (**Figure 1A**). The ovaries exhibited distinct borders, well-developed follicles and corpora lutea, and no signs of fibrotic or hemorrhagic tissues. Besides, no differences in size were observed (control: 4.2 mm^2^ ± 0.2; enriched: 4.3 mm^2^ ± 0.2).

The number of primordial, primary, preantral, antral, and atretic follicles was analyzed and no differences were detected between the groups (**Figures 1B–F**). However, ovaries from enriched females presented more corpora lutea than control females (p=0.017) (**Figures 1G, H**). Also, the size of the corpora lutea was larger in enriched mothers (p=0.049) (**Figure 1I**).

**Figure 2:**
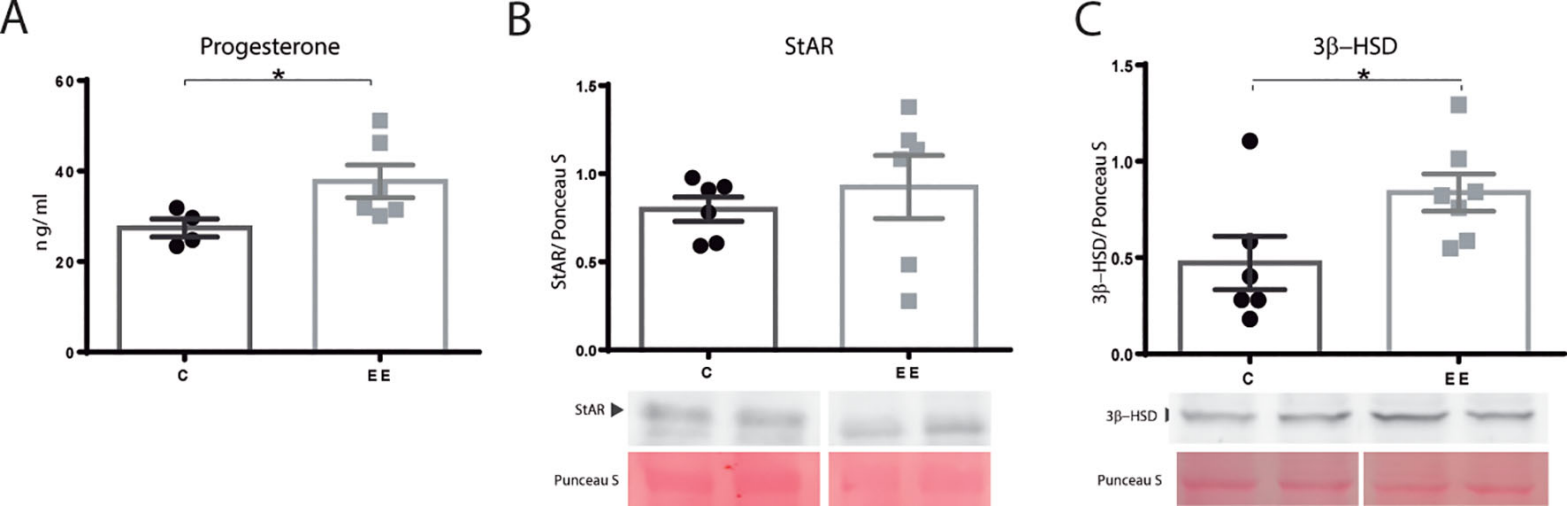
Progesterone levels and steroidogenic enzymes. **(A)** Progesterone serum levels. N= 4 control and 6 enriched environment animals. **(B)** StAR and **(C)** 3β-HSD protein levels N=6 control and 7 enriched environment animals. One ovary per animal was analyzed for western blot. C, control; EE, enriched environment. Results are expressed as mean ± s.e.m. T-test. *Significant statistical differences.

Subsequently, the progesterone level was determined in maternal serum. Enriched females presented higher progesterone levels than the control group (p=0.038) (**Figure 2A**).

The ovarian levels of proteins involved in steroidogenesis were determined by western blot. StAR, a rate-limiting protein in steroid production, did not change its expression (p=0.066) (**Figure 2B**). Nevertheless, the level of 3β-HSD, responsible for progesterone synthesis, was found to be elevated in enriched mothers (p=0.049) (**Figure 2C**).

## 4 Discussion

In the present study, we demonstrated that female mice exposed to an enriched environment present more corpora lutea, higher progesterone levels, and increased protein expression of 3β-HSD on day 7 of gestation. These results suggest that maternal lifestyle can affect the physiology of the ovary and modulate the production of progesterone, a crucial hormone for maintaining pregnancy.

Results in other animal models support our observations. It has been reported that housing conditions could affect ovarian physiology. Social interaction modulates progesterone levels in non-pregnant mice (13). Also, it was reported that the environment in which gilts are housed modulates the mRNA levels of enzymes involved in steroidogenesis, like StAR and 3β-HSD (14).

In line with these observations, in the present work, we report that enriched females exhibit a higher number of corpora lutea compared to the control group. In mice, the corpus luteum is the principal source of progesterone throughout pregnancy, and it is well-described that progesterone is necessary for maintaining gestation (1). Previously, we reported that enriched females exhibit a higher pregnancy rate than control mothers (7). We described that maternal environmental enrichment improves the maintenance of gestation and prevents pregnancy loss. Therefore, we hypothesize that the higher progesterone serum levels could explain this increase in pregnancy rate, as progesterone is essential to prevent the contraction of the uterine muscles that could interfere with embryo implantation and early gestation. Also, progesterone stimulates decidualization and the secretory activity of the uterine glands necessary for the developing embryo (15).

Additionally, steroid hormones promote uterine vascular remodeling and modulate uterine artery contractility, impacting uterine blood flow during pregnancy (16). Specifically, uterine arteries express progesterone and estrogen receptors and are reactive to these steroids (17). Previously, we observed that these steroids promote the acquisition of the human first-trimester trophoblast endovascular phenotype (18). Also, progesterone stimulates nitric oxide synthase activity and, specifically, endothelial nitric oxide synthesis in endothelial cells (19). Recently, we reported that the maternal environmental enrichment protocol used in the present work induces changes in the micro and macrovasculature that are paralleled by the modulation of molecules involved in vascular processes, like VEGF-A, nitric oxide, and endoglin (7). Therefore, the increase in serum progesterone levels accompanies the stimulation of the vascular adaptations previously observed in enriched females.

Progesterone metabolism could be regulated, among others, by StAR and 3β-HSD proteins. For its part, StAR plays a pivotal role in steroidogenesis, as it is the rate-limiting step in steroid production. It is responsible for transporting cholesterol to the inner membrane of the mitochondria, where the enzymes involved in steroidogenesis are located. This protein is primarily expressed by granulosa and theca cells in the follicle (20). In our work, no differences in the levels of this protein were detected. However, enriched females presented higher protein levels of 3β-HSD. This enzyme converts pregnenolone into progesterone and is expressed by granulosa and theca cells in the follicle and luteal cells in the corpus luteum (20). The increase in the protein levels of 3β-HSD could explain the increment in progesterone levels observed in the present work.

The modulation of ovarian physiology is regulated by the hypothalamus and the limbic system, which are sensitive to external stimuli. They respond by releasing neurotransmitters that modulate the hypothalamus-pituitary-gonadal axis. This axis regulates reproductive processes by hormones that affect the ovaries and stimulate follicular development. The intricate interplay between the hypothalamus, the limbic system, and the hypothalamus-pituitary-gonadal axis is vital to maintaining proper reproductive function (21). Another possible mechanism linking how changes in the environment affect ovarian physiology could be the regulation of neurotrophins such as VIP or BDNF. The nervous system produces neurotrophins in response to environmental stimuli. Concerning this, neurotrophin levels are modified by exercise and other lifestyle-related factors (22). Moreover, it is reported that they participate in follicular development, oocyte maturation, ovulation, and maintenance of the corpus luteum. Also, they contribute to the stimulation of steroid hormone production in the ovaries (23, 24). Therefore, we propose that these mechanisms might be involved in the modulation of the ovarian physiology in our model.

In conclusion, our results demonstrate that the enrichment of the maternal environment regulates ovarian physiology. The increase in corpora lutea number and size, as well as progesterone serum levels, provide support for the idea that exposure to an enriched environment exerts a protective role in gestation, preventing embryo loss. Even considering the limitations of an animal model, the positive effects of the enriched environment support the idea that a maternal enrichment protocol could be recommended for women seeking pregnancy. This non-pharmacological intervention might be advantageous, particularly for women undergoing fertility treatments.

## Data Availability

The raw data supporting the conclusions of this article will be made available by the authors, without undue reservation.
